# Selective Impairments in Fine Neural Tuning for Print in Chinese Children with Developmental Dyslexia

**DOI:** 10.3390/brainsci13030379

**Published:** 2023-02-22

**Authors:** Licheng Xue, Jing Zhao, Xuchu Weng

**Affiliations:** 1 Key Laboratory of Brain, Cognition and Education Sciences, Ministry of Education, Guangzhou 510599, China; 2 Institute for Brain Research and Rehabilitation, South China Normal University, Guangzhou 510599, China; 3 Jing Hengyi School of Education, Hangzhou Normal University, Hangzhou 311121, China; 4 Zhejiang Philosophy and Social Science Laboratory for Research in Early Development and Childcare, Hangzhou Normal University, Hangzhou 311121, China; 5 Zhejiang Key Laboratory for Research in Assessment of Cognitive Impairments, Hangzhou 311121, China

**Keywords:** developmental dyslexia, N1, neural tuning, visual word processing, Chinese

## Abstract

Neural tuning for print refers to differential neural responses (e.g., the N1 component of event-related potentials) to different orthographic forms and other visual stimuli. While impaired neural tuning for print has been well established in dyslexic children who read alphabetic scripts, it remains unclear whether such effects exist in dyslexic children who read Chinese, which dramatically differs in visual and linguistic characteristics from alphabetic words. To fill this gap, we examined two levels of the neural tuning for print: coarse tuning (i.e., false character vs. stroke combination), and fine tuning (i.e., sub-lexical tuning: pseudo character vs. false character; and lexical tuning: real character vs. pseudo character). Using the event-related potential technique, we examined 14 typically developing children and 16 dyslexic children who were screened from 216 nine-year-old children in the third grade. For typically developing children, we observed both coarse and sub-lexical tuning. Critically, for dyslexic children, we found stronger N1 for false character than for stroke combination, suggesting intact coarse tuning, but a reduced N1 difference between false character and pseudo character, suggesting impaired sub-lexical tuning. These results clearly show selective impairments in fine neural tuning at the sub-lexical level in Chinese dyslexic children. Our findings may be associated with unique features of Chinese characters.

## 1. Introduction

Developmental dyslexia (DD) is the most common learning disability and affects 5 to 17% of the population worldwide. DD is a reading difficulty despite conversational education and adequate intelligence [1,2]. Research on the neural basis of reading difficulties can help to establish biomarkers for the diagnosis and intervention of DD [3]. Neural tuning for print over the occipitotemporal area plays a key role in fast visual word reading [4,5,6]. We thus focused on the neural tuning for print in Chinese DD children.

Extensive studies including dyslexic children who read alphabetic scripts have consistently detected impairments in their neural tuning for print [7]. Using the EEG N1 (N170) component as a measure, these impairments are mainly manifested at two levels: One is the impaired *coarse* neural tuning for print—that is, compared with typically developing (TD) children, DD children show reduced N1 difference between words or letter strings and symbol strings (e.g., [8,9,10,11]). The other is the impaired *fine* neural tuning—that is, reduced N1 differences among words, pseudo words, and irregular letter strings in DD children compared with TD children (e.g., [8,9,12]).

It is noteworthy that almost all previous studies on neural tuning for print have been conducted on dyslexic children using alphabetic scripts. Compared to alphabetic words, Chinese characters fundamentally differ in their visual and linguistic characteristics [13] (see below for a discussion). Furthermore, recent research suggests different neural foundations in dyslexia across alphabetic and Chinese scripts (e.g., [14,15]). Therefore, it is crucial to examine whether the above impairments of neural tuning for print also exist in dyslexic children who learn to read Chinese. To the best of our knowledge, no such study has been conducted.

**Figure 1:**
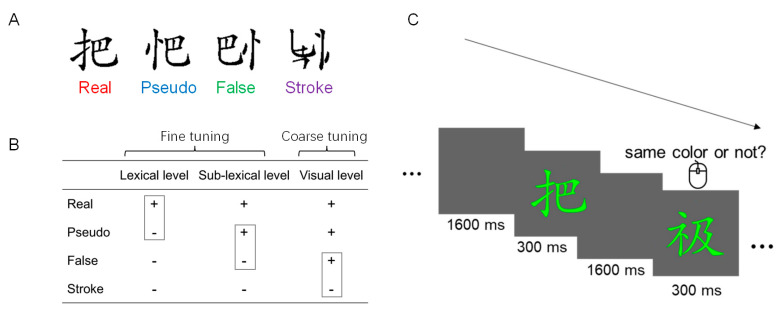
(**A**) Examples of stimuli. (**B**) Summary of the neural tuning for print levels. (**C**) The schematic depiction of the color-matching task.

Therefore, we aimed to characterize neural tuning for print in Chinese DD children. For this purpose, we used three types of characters (i.e., real, pseudo, and false characters) and stroke combinations (see Figure 1A for examples). As shown in Figure 1B, we focused on the coarse tuning (i.e., false character vs. stroke combination) and the fine tuning (i.e., sub-lexical level: pseudo character vs. false character; lexical level: real character vs. pseudo character). To minimize task-demand linguistic processing, we used a content-irrelevant color-matching task (see [16] for detailed discussion). Additionally, to reduce potential confounding related to the compensatory effect [17], we investigated dyslexic children in the early acquisition stage, which is also a critical time window for the diagnosis of dyslexia in mainland China (i.e., 9 years, third grade; see [2]).

According to previous N1 studies on Chinese TD children [18], as expected, TD children would show coarse tuning as well as sub-lexical fine tuning. In alphabetic children, because of systematic mapping between orthographic forms and sounds, the acquisition of grapheme–phoneme correspondence should be crucial for the emergence of coarse tuning for print [19]. Indeed, several studies have consistently found impairments in coarse tuning in DD children who read alphabetic scripts [8,9,10,17]. In contrast, Chinese characters map onto phonology at the syllable level, and there is essentially no grapheme–phoneme correspondence [20]. It has been shown that orthography, but not phonology, is the primary driver of neural tuning for print [21]. Chinese TD children (7 years, first grade) have acquired low levels of orthographic regularity knowledge (i.e., the ability to distinguish characters from stroke combinations in a lexical decision task) after they start learning to read and, in turn, exhibit the coarse tuning [18].

Furthermore, children’s knowledge of orthographic regularity is closely related to their reading skills [18,22]. For Chinese DD children aged 9 in the third grade, they may have acquired the low-level orthographic regularity knowledge to distinguish characters from stroke combinations, because of their comparable reading skills to TD children aged 7 in the first grade. Therefore, we expected that DD children would show the coarse tuning for print, reflected in stronger N1 for false character than for stroke combination. In contrast, due to a lack of high levels of orthographic regularity knowledge at this age [23,24], we expected that the fine tuning for print would be impaired in dyslexic children’s brains. Specifically, we expected that DD children would show a reduced N1 difference in the comparison of false characters and pseudo characters. In summary, we expected that the coarse tuning for print would be intact, but the fine tuning would be impaired in Chinese DD children.

## 2. Methods

The research protocol was approved by Hangzhou Normal University’s ethical committee (IRB #20130301h), and written informed consent was obtained from all participants and their parents. The protocols adhered to the Helsinki Declaration.

### 2.1. Participants

Two-hundred and sixteen third-grade children were screened. The tools were the Chinese character recognition test [25] and Standard Combined Raven’s Test (CRT [26]; see the next section for details). Based on the procedure and criteria, which have been widely used in the previous studies of Chinese dyslexic children (e.g., [14,27,28,29,30,31]), the criteria for screening and justifying dyslexic children in the present study were as follows: (a) Chinese character recognition score one standard deviation or more below the grade-level average, (b) with normal non-verbal intelligence (IQ > 85), (c) no neurological or psychiatric diseases reported by parents or teachers, and normal or corrected-to-normal visual acuity. A total of 19 children met these criteria and consented to take part in the study, but 3 participants were excluded because of noise in their EEG recordings.

Chronological age-matched typically developing (TD) children were selected in the same grade at the same school. Their Chinese character recognition scores were at or above the grade average. Fifteen children consented to participate in the study, but one of them quit during testing.

The participants were all native Mandarin speakers and right-handed. The results shown here are based on a total of 16 dyslexic children (DD; 8 boys and 8 girls) and 14 age-matched controls (TD; 7 boys and 7 girls). Table 1 illustrates the children’s age, CRT, and Chinese character recognition scores. The mean age of the two groups was matched (*t*(28) < 1.0, *p* = 0.37), as was the mean of the CRT scores (*t*(28) < 1.0, *p* = 0.58). By contrast, the TD group had a significantly higher mean score of Chinese character recognition than the DD group (*t*(28) = 12.21, *p* < 0.001).

### 2.2. Cognitive Assessments

#### 2.2.1. Chinese Character Recognition Test

The Chinese character recognition test is a widely used tool in Chinese dyslexia research [25]. The test contains a list of 150 Chinese characters of increasing difficulty. Children are instructed to read out these characters one by one during the test. The score of the test has been shown to be a good indicator of Chinese children’s reading ability in previous studies [32,33,34]. When the score is one standard deviation below the grade-level average, the child is considered to have reading difficulties and may have dyslexia [14,30,31,32,33].

#### 2.2.2. Standard Combined Raven’s Test

This test is widely used to assess the non-verbal intelligence in Chinese dyslexia research [14,30,34]. This test has high reliability (the retest reliability is 0.95, and the split-half reliability is 0.97) and validity (significant correlation coefficient with the full scale of the Wechsler Intelligence Test = 0.56) [35]. In this test, children are presented with a mutilated picture and six options. They are instructed ‘You see, in the picture above, there is a piece missing, so please choose the best piece to fill in this picture from the following six small pieces. Please find out which piece matches best’. This test contains five sections (A to E). Each section consists of 12 items. All items in Sections A, B, and C need to be answered. In Sections D and E, when three consecutive items are incorrect, this section is stopped. One point is awarded for one correct answer. Raw scores are transferred to standard scores according to the norm.

#### 2.2.3. Materials

We adopted three stimulus types of characters (i.e., real, pseudo, false) and stroke combinations (see Figure 1A). All characters contained two radicals that were left- and right-structured. The average number of strokes was well matched. Half of the real characters were at a low frequency of occurrence (1 to 5 per million), and half of the characters were at a high frequency (400 to 4500 per million), according to the *Modern Chinese Character Frequency Dictionary* (1985). Using the same set of radicals as that in real characters, we constructed pseudo and false characters. For pseudo characters, the position of the two radicals was legal. By contrast, for false characters, the two radicals were arranged in their illegal positions. A stroke combination was composed of a false character by disrupting the strokes of its radicals, but keeping the left and right structures. Each stimulus type consisted of 36 stimuli (6 as targets), each of which was either green, red, or yellow. Each color appeared the same number of times across stimulus types.

#### 2.2.4. Procedure

There were four blocks, each of which included 108 trials. All stimuli were presented randomly, but two successive stimuli of the same type were prohibited. Each stimulus was presented in the center of the screen with a gray background. In each trial, the stimulus duration was 300 ms. The ISI was randomized across trials, uniformly distributed among an array of 1450 ms, 1525 ms, 1600 ms, 1675 ms, and 1750 ms. To obtain enough trials for ERP averaging, all stimuli were presented three times. A similar procedure was used in previous studies [16,18,21,36,37,38,39]. There were 108 trials in each stimulus type (90 non-target trials). Participants were asked to press a key when they saw two stimuli of the same color appearing in succession (see Figure 1C). The response key was balanced across subjects.

### 2.3. Data Collection and Statistical Analysis

An elastic cap with 30 Ag/AgCl electrodes was used, which was montaged according to the extended 10–20 system [40]. A DC amplifier system from BrainAmp ExG with its BrainVision Recoder software (Brain Products GmbH, Gilching, Germany) was used to record the EEG signals. The ground node was AFz. The online reference electrode was located between Cz and CPz. Additionally, to correct eye-blink artifacts, we recorded both electrooculograms (EOGs). A value of 5 kΩ was adopted as the impedance criterion. The signals were AC-amplified between 0.1 and 100 HZ, sampling at 1000 Hz.

Offline data were filtered twice, with a 0.1 Hz/high-pass filter, and then with a 30 Hz/low-pass filter. Before averaging, we scanned each recording epoch for artifacts manually. We corrected trials for eye movement artifacts offline through using the ICA procedure [41]. Trials were then epoched with a 100 ms pre-stimulus period and a 700 ms post-stimulus period. The 100 ms before the stimulus onset was used for baseline correction. False positive responses in each type were excluded from the further analyses. Epoch signals that exceeded ±100 µV were rejected. Three pairs of channels were selected (P7, O1, and TP9 as the left ROIs, and P8, O2, and TP10 as the right ROIs). These channels were the topographic maxima in the negative field on the occipitotemporal area over both hemispheres, usually used to measure N1 in many previous studies [18,36,37,39,42]. The time windows of P1 and N1 were determined by the first and second components of global field power (GFP) [43]. The peak amplitude of each selected channel was detected in the time windows of P1 and N1, and then averaged by hemisphere. The peak latencies of both components were represented by the P7/P8 electrodes.

## 3. Results

**Figure 2:**
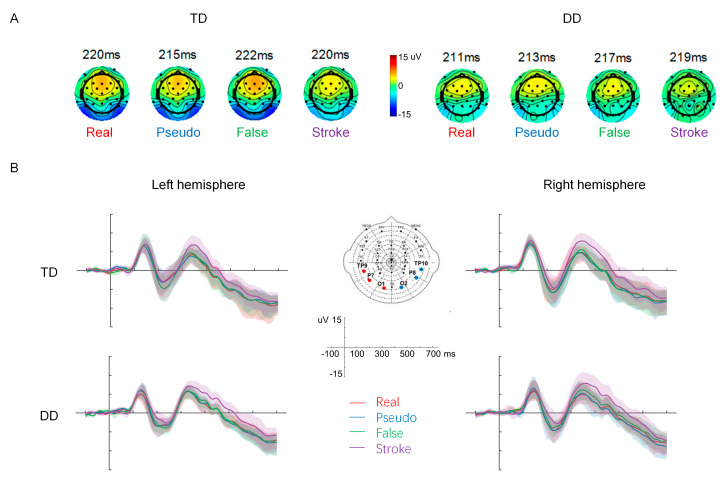
(**A**) Topographic maps of 4 stimulus types at N1 peaks in the TD and DD groups. (**B**) ERP waveforms of both ROIs with 95% confidence intervals (shaded areas, bootstrap method) in the two groups.

Figure 2 illustrates the topographic maps and the occipitotemporal ROI ERP waveforms of the four stimulus types separately for the TD and DD children. A robust N1 component was observed in each stimulus type in each group of children.

P1 and N1 were mainly analyzed. According to previous studies, P1 is usually associated with the processing of low-level visual features, while N1 is recognized as an electrophysiological marker reflecting the neural tuning for print [5]. ANOVA was used to analyze the data, factorized with two within-subject factors—stimulus type (i.e., real, pseudo, false, stroke) and lateralization (left vs. right)—as well as one between-subject factor, i.e., reading group (TD vs. DD).

Neither DD children nor TD children showed robust differences among the four stimulus categories on the P1 component (see the Supplementary Materials for detailed results). This is not surprising, given that the low-level visual features were matched among the four stimulus categories.

### 3.1. N1 Amplitude for Four Types of Stimuli in TD and DD Children

**Figure 3:**
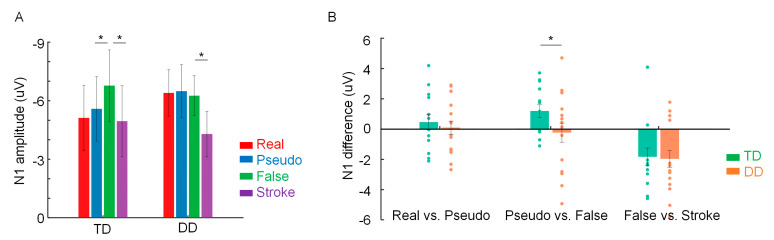
(**A**) Mean N1 amplitudes for the four stimulus types in the left hemisphere in TD and DD children. (**B**) Bar plots of mean N1 difference in the left hemisphere in each group of children. Error bar denotes SE; * refers to *p* < 0.05.

Figure 3A illustrates the mean N1 amplitude evoked by the four types of stimuli in the left hemisphere in both groups of children.

### 3.2. Intact Coarse Neural Tuning for Print in Chinese DD Children

The results showed that the main effect of stimulus type was significant (*F*(3, 84) = 12.69, *p* < 0.001, *η^2^* = 0.31). The other main effect was not significant, with *F*-values < 1. The stimulus type by reading group interaction was significant (*F*(3, 84) = 3.77, *p* = 0.01, *η^2^* = 0.12). No other significant interaction was found—for stimulus type by lateralization (*F*(3, 84) = 2.46, *p* = 0.07, *η^2^* = 0.08); for reading group by lateralization (*F*(1, 84) = 2.26, *p* = 0.14, *η^2^* = 0.08); or for stimulus type by lateralization by reading group (*F*-values < 1). Results in the further analysis revealed stronger N1 for orthographic stimuli than for stroke combination in DD children (all *p*-values < 0.01). Similarly, TD children showed stronger N1 responses to false characters relative to stroke combinations (*p =* 0.02). To sum up, these results clearly suggest that the coarse tuning may be intact in dyslexic children who learn to read Chinese.

### 3.3. Impaired Fine Neural Tuning for Print in Chinese DD Children

The aforementioned results showed that TD and DD children showed stronger N1 for orthographic stimuli than for stroke combination (i.e., the coarse tuning for print). As discussed above, the theoretical hypothesis on the fine tuning for print was mainly about N1 responses to orthographic stimuli. Therefore, in the next analysis we focused on N1 responses to the orthographic stimuli (real, pseudo, false), using a 3 × 2 × 2 three-way ANOVA. The main results revealed a significant stimulus type by reading group interaction (*F*(2, 56) = 4.47, *p* = 0.02, *η^2^* = 0.14), and the stimulus type by lateralization interaction was also significant (*F*(2, 56) = 3.79, *p* = 0.03, *η^2^* = 0.12). The three-way interaction of stimulus type by lateralization by reading group was *F*(2, 56) < 1. A hemispheric difference in N1 tuning for print has been widely reported in previous studies (see [5,16,18,39] for an overview). To examine our hypothesis more clearly, we conducted planned post hoc simple effects analysis for the three-way interaction.

The main results pertaining to our hypothesis are shown in Figure 3A, illustrating different patterns of N1 for real, pseudo, and false characters in the left hemisphere between TD and DD children. For the TD group, we observed an increased N1 response to false characters relative to pseudo or real characters (for false vs. pseudo, *p* = 0.04; for false vs. real, *p* = 0.01), while there was no significance in the comparison of real characters and pseudo characters (*p* = 0.99). By contrast, for DD children, the N1 difference was not significant among these three stimulus types (all *p*-values *>* 0.99). Over the right hemisphere, no significant N1 difference was found among these stimulus types in either TD or DD children (all *p*-values *>* 0.14). In summary, these results suggest that the fine tuning may be impaired in Chinese dyslexic children.

### 3.4. N1 Amplitude Difference between Two Stimulus Types in TD and DD Children

As shown in Figure 3B, in the left hemisphere, the N1 difference (false vs. stroke) was found in both the TD and DD groups, while the N1 difference (real vs. pseudo) largely varied even in TD children. Notably, N1 (pseudo vs. false) widely existed in most of the TD children, but not in DD children.

The results of the independent-samples *t*-test (one-way) revealed that neither the false vs. stroke nor the real vs. pseudo N1 difference was significant between the TD and DD groups (for false vs. stroke, *t* (28) = 0.57, *p* = 0.29; for real vs. pseudo, *t* (28) = 0.17, *p* = 0.43). Critically, the N1 difference (pseudo vs. false) was significantly reduced in DD children compared with TD children (*t* (28) = 1.83, *p* = 0.04). In summary, the results suggest that the coarse tuning may be comparable in DD and TD children, whereas the fine tuning may be reduced in DD children compared with TD children.

In addition, neither DD children nor TD children showed significant differences among the four stimulus types in N1 latency (see the Supplementary Materials for detailed results).

## 4. Discussion

The impaired neural tuning for print has been extensively studied and consistently demonstrated in alphabetic dyslexic children. Chinese characters are dramatically different from alphabetic words in terms of visual and linguistic characteristics. However, the characteristics of the neural tuning in Chinese dyslexic children remain unclear. We addressed this issue by comparing the patterns of N1 responses to stimuli with different orthographic regularities. We observed both coarse and fine tuning in TD children. Critically, DD children produced strong N1 for false characters relative to stroke combinations, whereas false characters evoked similar N1 responses to pseudo characters. Our findings suggest that the fine neural tuning is selectively impaired in Chinese DD children.

Our first main finding was that DD children produced increased N1 responses to false characters compared with stroke combinations; furthermore, the N1 difference between these two stimulus types was similar in both DD and TD children. These results indicate that the coarse neural tuning for print may be intact in Chinese dyslexic children. In contrast, previous studies found that the coarse tuning was impaired in alphabetic dyslexic children. Specifically, compared with TD children, alphabetic dyslexic children showed reduced N1 differences between letter and symbol strings [8,9,10,44]. One plausible explanation is that the drastic linguistic differences between Chinese characters and alphabetic words led to this discrepancy between the current findings and those in alphabetic children. In alphabetic words, orthographic forms such as letters systematically map to sounds. Indeed, studies on alphabetic readers show that the coarse tuning for print appears quickly after acquiring the grapheme–phoneme correspondences in preschool children [19]. The ability of phonological decoding is impaired in alphabetic dyslexic children even after learning to read [11,45]. Therefore, the coarse tuning appears to be more vulnerable to impairment in alphabetic dyslexic children. In contrast, visual forms are mapped to phonological forms relatively arbitrarily in Chinese [20]. Orthography rather than phonology mainly drives the neural tuning for print (see [21] for a detailed discussion). TD children aged 7 in the first grade have acquired low-level orthographic regularity knowledge to distinguish characters from stroke combinations, leading to the emergence of coarse tuning for print [18]. Results from previous studies have shown that children’s orthographic regularity knowledge is closely related to their reading skills [22,38]. Due to their having comparable reading skills to TD children aged 7 in the first grade, DD children aged 9 in the third grade may have acquired this low-level orthographic regularity knowledge. Therefore, Chinese DD children produced the coarse tuning in our study. It is also possible that task-demand difference has an impact. While previous studies usually employed a content-relevant task, our study adopted a content-irrelevant task (the color-matching task). The coarse tuning for print may be more vulnerable in the content-relevant task, which is strategically more dependent on the phonological processing [16]. However, this issue needs further studies to clarify it. Through using various types of tasks measuring the same group of dyslexic children, further studies may advance our understanding of the coarse tuning for print in dyslexic children.

Our second key finding is that DD children produce similar N1 responses to pseudo and false characters, and the N1 difference between these two stimulus types was significantly reduced relative to TD children. These results suggest that the fine neural tuning in Chinese DD children was impaired at the sub-lexical level. In agreement with our results, previous studies in alphabetic dyslexic children have consistently found reduced N1 difference between pseudo words and false words [8,9,12]. Therefore, there may be a universal impaired neural tuning for print in dyslexic children across different writing systems. This is because acquisition of knowledge of orthographic regularity is a fundamental and central part of reading, not only in alphabetic scripts, but also in Chinese script. In our study, the pseudo characters were composed of two radicals that never appear together in the lexicon, but each radical occurred at a regular position, whereas these two radicals were placed in illegal positions in the false characters. Due to lack of phonology or semantics of their own, the difference between pseudo characters and false characters was mainly in orthographic regularity (more precisely, the radical position). Previous behavioral studies have shown that the high levels of orthographic knowledge are impaired in Chinese dyslexic children [46]. For instance, dyslexic children showed significantly decreased accuracy in responding to false characters in a lexical decision task, compared with TD children [23,24]. Orthography plays a key role in driving the fine neural tuning for print [21]. Therefore, we observed that the fine tuning was impaired in dyslexic children.

Our findings could be generalized to alphabetic DD children and provide an alternative explanation for the impaired neural tuning for print in alphabetic dyslexic children. As discussed previously, due to the systematic grapheme-to-phoneme conversion, pseudo words not only conform to orthographic rules, but are also pronounceable. Specifically, pseudo words and false words differ in both orthographic regularity and phonology in alphabetic scripts. Therefore, the reduced N1 difference between false and pseudo alphabetic words may be due to defects in either orthographic or phonological processing. In contrast, the unique properties of Chinese characters can tease apart orthographic and phonological properties. In addition, by using a content-irrelevant color-matching task, the involvement of potential phonological processing was further reduced. In this context, we still observed significantly reduced N1 differences between pseudo and false characters in DD children. Our results indicate that the impaired fine tuning is more likely due to defects in orthographic (but not phonological) processing, regardless of script type. However, this inference still needs to be verified in future studies—for example, by using various types of tasks and directly comparing the N1 responses to pseudo and false words in alphabetic DD children.

Consistent with previous studies on alphabetic dyslexic children, we observed that DD children produced similar N1 responses to real characters relative to pseudo characters. Interestingly, the N1 difference of real vs. pseudo characters was also absent in TD children. One possible interpretation is that the neural tuning for real characters relative to pseudo characters develops slowly as children learn to read, even for TD children. Consistent with this idea, an earlier study of Chinese TD children found that children showed a greater N1 difference between real and pseudo characters with more reading experience (i.e., at the age of 11, but not 9) [18]. However, this interpretation needs further verification. Future studies on TD and DD children in the later stages of learning to read would advance our understanding about the neural tuning for print at the lexical level in dyslexic children.

While beyond the scope of this study, several issues need further research to address them. First, it is necessary to delineate the sources of the impaired neural tuning in the brain. The EEG technique adopted in our study has limited spatial resolution. Techniques with high spatial precision, such as fMRI, would further delineate the sources that the impaired neural tuning for print stems from. Second, previous research has found increased responses in frontal areas as DD children read words, suggesting compensatory effects [47]. However, such compensation may be not necessarily restricted to frontal regions [48]. To minimize the potential influence of the compensation, we investigated DD children in the early stages of reading acquisition. However, we know little about whether the compensatory brain activity also occurs in posterior brain areas. By examining older children and adults with DD, further studies may provide evidence for understanding the impairment and compensation involved in neural tuning for print.

## 5. Limitations

Before closing our discussion, we would like to note a few limitations of the present study. First, as no language information was needed to complete the color-matching task, the features of the neural tuning for print in DD children in language-relevant tasks remain unclear. Second, we only tested one age group of DD children. To further characterize the developmental profile and examine whether the compensation is involved in the neural tuning for print, future studies should expand the range of ages. Third, the sample size of the present study was small. Future studies need to use larger sample sizes to verify our findings.

## 6. Conclusions

We observed that both TD and DD children showed similar N1 responses for real characters relative to pseudo characters, while they showed increased N1 responses to false characters relative to stroke combinations. However, TD children showed increased N1 responses for false characters compared with pseudo characters, whereas DD children produced reduced N1 differences between these two stimulus types. These results show that the fine neural tuning for print is selectively impaired in dyslexic children who learn to read Chinese. Our findings may be related to unique features of Chinese characters, but they may also contribute to understanding impairments of neural tuning in alphabetic dyslexic children from the perspective of script universality.

## Figures and Tables

**Table 1:** Mean age, CRT score, and reading performance.

Group	Age (Years)	CRT Score	Reading Score
TD	9.05 (0.38)	108.07 (16.95)	114.57 (12.04)
DD	8.95 (0.25)	105.25 (9.80)	66.81 (9.37)

Note: values in parentheses are standard deviations.

## Data Availability

The data, experimental procedures and analysis scripts can be obtained by contacting the corresponding author.

## Supplementary Materials

The following supporting information can be downloaded at: https://www.mdpi.com/article/10.3390/brainsci13030379/s1.
