# Supplementary material for: Selective Impairments in Fine Neural Tuning for Print in Chinese Children with Developmental Dyslexia
Source: Brain Sci. 2023 Feb 22;13(3):379. doi: 10.3390/brainsci13030379 (PMC10046482; doi:10.3390/brainsci13030379)
Supplement: Supplementary file 1 [file brainsci-13-00379-s001.zip › brainsci-2242681-supplementary.pdf]

Supplementary Materials for

Selective impairments in fine neural tuning for print in Chinese children with developmental dyslexia

Results

P1 component

P1 amplitude

Neither main effect of the three-way ANOVA was significant, for Stimulus Type, F (3, 84) =1.19, p=0.32, $\eta_p^2 = 0.04$; for Lateralization, F (1, 28) <1; for Reading Group, F (1, 28) =1.22, p=0.28, $\eta_p^2 = 0.04$. In addition, neither the interaction of the three-way ANOVA was significant, for Stimulus Category by Reading Group, F (3, 84) =1.02, p=0.38, $\eta_p^2 = 0.04$; for the other interactions, all *F-values* <1.

P1 latency

Results in the three-way ANOVA showed that the main effect of Stimulus Type showed a trend, F (3, 84) =2.45, p=0.09, $\eta_p^2 = 0.08$. The interaction of Lateralization by Reading Group was significant, F (1, 84) =4.76, p=0.04, $\eta_p^2 = 0.15$. However, the further analysis did not find significant difference across the four stimulus types (all *p-values*>0.1). In P7 electrode, TD children showed a trend in longer P1 latency than DD children (p=0.07). TD children showed a trend in shorter latency in P7 electrode relative to P8 electrode (p=0.08). Neither the main effect of Lateralization nor Reading Group was significant, all *F-values* <1. The interaction of the three factors

was not significant, for Stimulus Category by Lateralization, F (3, 84) =2.02, p=0.13, η_p^2 = 0.07; for the other interactions, all *F-values* <1.

N1 component

N1 latency

Table S1 shows the N1 peak latency for four types of stimuli on the P7 and P8 channels in TD and DD groups.

Table S1 N1 peak latency (msec) in the TD and DD groups

	P7 electrode				P8 electrode			
Group	Real	Pseudo	False	Stroke	Real	Pseudo	False	Stroke
TD	220 (6)	215 (6)	222 (5)	219 (5)	214 (5)	212 (5)	213 (5)	219 (5)
DD	211 (5)	213 (5)	217 (5)	219 (5)	221 (4)	222 (4)	219 (4)	221 (4)

Notes: In the parentheses are SEM.

Results in a similar three-way ANOVA revealed the main effects of Stimulus Type was significant, F (3, 84) =3.00, p=0.04, η^2= 0.10. No other significant main effect was found, all *F-values*<0.1. There was a trend in the Stimulus Type by Lateralization interaction, F (3, 84) =2.29, p=0.09, η^2= 0.08. Neither Reading Group by Lateralization interaction, F (1, 28) =2.49, p=0.13, η^2= 0.08, nor Reading Group by Stimulus Type interaction, F <1, was significant. There was a trend in Stimulus Type by Lateralization by Reading group interaction, F (3, 84) =2.43, p=0.07, η^2= 0.08. Further analysis showed that TD children showed a trend in shorter N1 latency for

pseudo characters than for stroke combination (p=0.05) on P8 electrode, while DD children showed a similar effect on P7 electrode (p=0.09).

Stimuli list

Real character
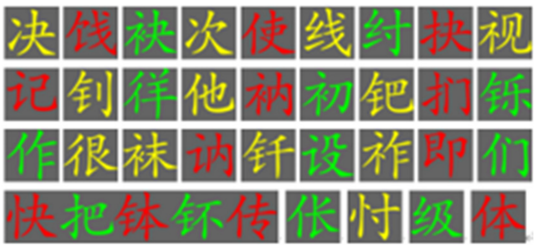
Pseudo character
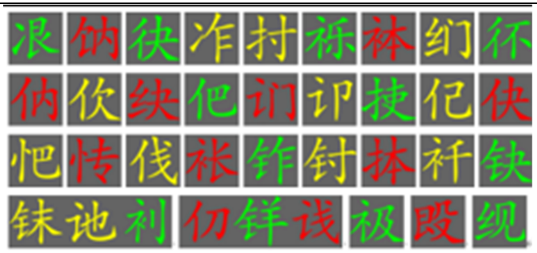
False character
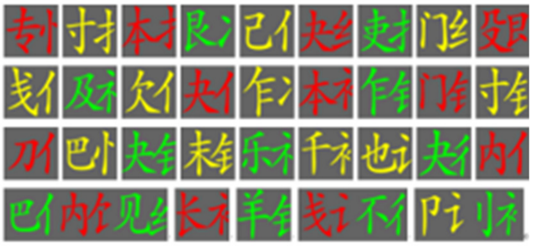
Stroke combination
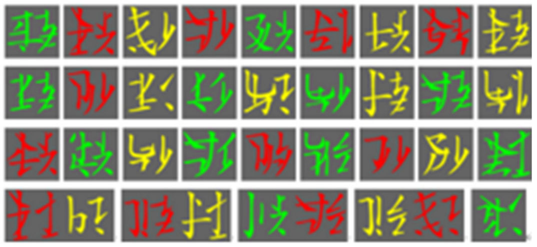
